# Synthesis and Characterization of Novel Nanoparticles of Lithium Aluminum Iodate LiAl(IO_3_)_4_, and DFT Calculations of the Crystal Structure and Physical Properties

**DOI:** 10.3390/nano11123289

**Published:** 2021-12-03

**Authors:** Rihab Chikhaoui, Zoulikha Hebboul, Mohamed Abdelilah Fadla, Kevin Bredillet, Akun Liang, Daniel Errandonea, Sandrine Beauquis, Ali Benghia, Jean Christophe Marty, Ronan Le Dantec, Yannick Mugnier, Enrico Bandiello

**Affiliations:** 1 Laboratoire Physico-Chimie des Matériaux (LPCM), Université Amar Telidji de Laghouat, BP 37G, Laghouat 03000, Algeria; rihabchikhaoui.26@gmail.com (R.C.); z.hebboul@lagh-univ.dz (Z.H.); 2 Laboratoire de Physique des Matériaux, Université Amar Telidji de Laghouat, BP 37G, Laghouat 03000, Algeria; ma.fadla@lagh-univ.dz (M.A.F.); benghia11@gmail.com (A.B.); 3 SYstème et Matériaux pour la MÉcatronique (SYMME), University Savoie Mont Blanc, F-74000 Annecy, France; kevin.bredillet@univ-smb.fr (K.B.); Sandrine.Beauquis@univ-smb.fr (S.B.); Jean-Christophe.Marty@univ-savoie.fr (J.C.M.); Ronan.le-Dantec@univ-smb.fr (R.L.D.); Yannick.Mugnier@univ-smb.fr (Y.M.); 4 Departamento de Física Aplicada—ICMUV—MALTA Consolider Team, Universitat de València, c/Dr. Moliner 50, 46100 Burjassot, València, Spain; akun2.liang@uv.es (A.L.); Daniel.Errandonea@uv.es (D.E.)

**Keywords:** precipitation, crystal structure, X-ray diffraction, scanning electron microscopy, optical spectroscopies, computer simulations

## Abstract

Here we report on the non-hydrothermal aqueous synthesis and characterization of nanocrystalline lithium aluminum iodate, LiAl(IO_3_)_4_. Morphological and compositional analyses were carried out by using scanning electron microscopy (SEM) and energy-dispersive X-ray measurements (EDX). The optical and vibrational properties of LiAl(IO_3_)_4_ have been studied by UV-Vis and IR spectroscopy. LiAl(IO_3_)_4_ is found to crystallize in the non-centrosymmetric, monoclinic *P*2_1_ space group, contrary to what was reported previously. Theoretical simulations and Rietveld refinements of crystal structure support this finding, together with the relatively high Second Harmonic Generation (SGH) response that was observed. Electronic band structure calculations show that LiAl(IO_3_)_4_ crystal has an indirect band gap $E_{\mathrm{gap}}=3.68$ eV, in agreement with the experimental optical band gap $E_{\mathrm{gap}}=3.43\left(3\right)$ eV. The complex relative permittivity and the refraction index of LiAl(IO_3_)_4_ have also been calculated as a function of energy, as well as its elastic constants and mechanical parameters. LiAl(IO_3_)_4_ is found to be a very compressible and ductile material. Our findings imply that LiAl(IO_3_)_4_ is a promising material for optoelectronic and non -linear optical applications.

## 1. Introduction

Inorganic nanoparticles usually exhibit interesting magnetic, electrical, and optical properties, including non-linear optical (NLO) features. Many methods for the synthesis of inorganic nanoparticles exist, encompassing physical, chemical, and mechanical processes. The choice of the synthesis method is dictated by the structure that has to be obtained which, in turn, depends on the foreseen applications [1,2]. In fact, the nanoparticle size and size-distribution, the surface area, the chemical composition, as well as the purity, shape, and surface structure of the nanoparticles, play key roles in the resulting functional properties. Broadly speaking, synthesis methods can be subdivided into two categories: constructive methods and destructive methods. The former consists of the build-up of materials from single atoms to clusters and to nanoparticles, by methods including sol-gel [3,4], spinning [5], chemical vapor deposition [6], pyrolysis [7], and biosynthesis [8]. Conversely, in destructive methods, the reduction of bulk material to nanometric particles takes place through mechanical milling [9], nanolithography [10], laser ablation [11], sputtering [12], and thermal decomposition [13].

Non-centrosymmetric inorganic nanoparticles exhibit non-linear optical features (NLO), with generation of several harmonics and possible up-conversion of signals [14,15]. Nanocrystals have also been developed extensively for potential biomedical applications, molecular sensing devices, diagnostic imaging, and localization at tumor sites [16,17]. Many different families of nanomaterials have been studied with the aim of enhancing medical and NLO applications [18,19,20]. Metal iodates are considered to be among the most interesting nanomaterials [21,22,23,24,25,26,27,28,29], not only for NLO but also because of their dielectric properties and their unusual bonding properties, related to the presence of an electron lone pair on the iodine atom [30,31,32,33,34,35,36,37,38].

Hydrothermal and solvothermal synthesis methods are typically the most promising approaches for the preparation of metal iodate nanomaterials. These methods can be combined with microwave heating to improve the quality and the reproducibility of the final products [14].

In the first part of this work, we describe for the first time a simple and inexpensive chemical route for the preparation of pure nanocrystals of LiAl(IO_3_)_4_, which is a novel and interesting material for optoelectronics and nonlinear optics. LiAl(IO_3_)_4_ nanocrystals have been synthetized using a soft-chemistry method, which takes advantage of the direct precipitation reaction between lithium iodate (LiIO_3_) and aluminum nitrate nonahydrate (Al(NO_3_)_3_·9H_2_O) from highly concentrated water solutions, without hydrothermal conditions. To the best of our knowledge, this is one of the first examples of such a simple chemical route for the preparation of iodate nanoparticles. The morphology of the final product was inspected by electron microscopy, and the average size of particles was measured by X-ray diffraction, using Le Bail fir refinement and the Williamson-Hall method (W-H). UV-Vis and infrared spectra were also measured and discussed. Preliminary results dealing with a crude estimation of the SHG efficiency of LiAl(IO_3_)_4_ were also included after comparison of the emitted signals from two colloidal suspensions, the reference sample being LiNbO_3_ nanocrystals of similar size. The optical band gap of LiAl(IO_3_)_4_ nanoparticles was measured experimentally by absorbance spectroscopy. The second part of the manuscript is devoted to the theoretical calculation of the structural model and physical properties of this new compound using the Density-Functional Theory (DFT).

## 2. Materials and Methods

### 2.1. Sample Preparation and Characterization

Reagents purchased from Sigma–Aldrich (Burlington, MA, USA), lithium iodate (LiIO_3_, 99.5%) and aluminum nitrate nonahydrate (Al(NO_3_)_3_∙9H_2_O, 98%), were used without further purification. Powder X-ray diffraction patterns (XRD) of the final product were obtained at ambient conditions using a Panalytical EMPYREAN MALVERN (Malvern, UK) powder diffractometer (Cu Kα_1_, 40 mA, 30 kV) with a 0.01° step size and an acquisition time of 6 s/step in the 17–85° 2θ range. The homogeneity and chemical composition of the sample were investigated by Scanning Electron Microscopy (SEM) using a Tescan Vega (Brno - Kohoutovice, Czech Republic) electron microscope system attached to a Peltier-cooled XFlashTM (Bruker AXS—Karlsruhe, Germany) silicon drift detector, model 410 M, for energy dispersive X-ray (EDX) analysis. Optical absorption spectra were recorded using a Jasco V-630 UV-Vis (Easton, MD, USA) spectrophotometer in the 190–1100 nm wavelength range. Infrared spectra (FTIR) were recorded on a Jasco FT/IR-4200 (Easton, MD, USA) setup between 1000 and 400 cm^−1^ using KBr pellets. Dynamic light scattering (DLS) measurements (Zetasizer Nano, Malvern Panalytical, Palaiseau, France) were used to assess the colloidal stability and aggregation state of the synthesized samples after re-dispersion in ethanol.

### 2.2. Ab-Initio Calculations

The performed ab-initio calculations were based on the DFT theory [39,40] to calculate the structural, electronic, mechanical, and linear optical properties, using the pseudopotential plane-wave (PP-PW) method as implemented in the Cambridge Sequential Total Energy Package (CASTEP) module of Material Studio [39]. The exchange correlation interaction was described by the generalized-gradient approximation (GGA), using the Perdew-Burke-Ernzerhof (PBE) parameterization [40], with a kinetic cut-off energy of 700 eV for the plane-wave expansions. The reciprocal space integration was performed using (6 10 6) *k*-point grids in the first Brillouin zone (BZ). The geometry optimization was performed using the Broyden–Fletcher–Golfard–Shannon (BFGS) algorithm. The ultra-soft pseudopotential was used within the following valence electronic configuration: Li: 1s^2^ 2s^1^; O: 2s^2^ 2p^4^; Al: 3p^1^ 4s^2^; and I: 5s^2^ 5p^5^. For band structure calculations, in addition to the PBE functional we have implemented the nonlocal hybrid functional HSE06 (Heyd–Scuseria–Ernzerhof) [41], for a more precise determination of gap energy.

## 3. Results and Discussion

### 3.1. Synthesis and XRD Characterization

**Figure 1:**
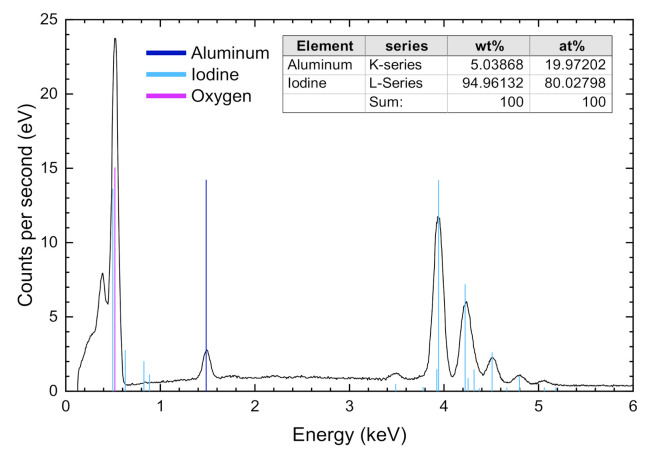
EDX elementary analysis of LiAl(IO_3_)_4_. No traces of impurities were found in our sample.

**Figure 2:**
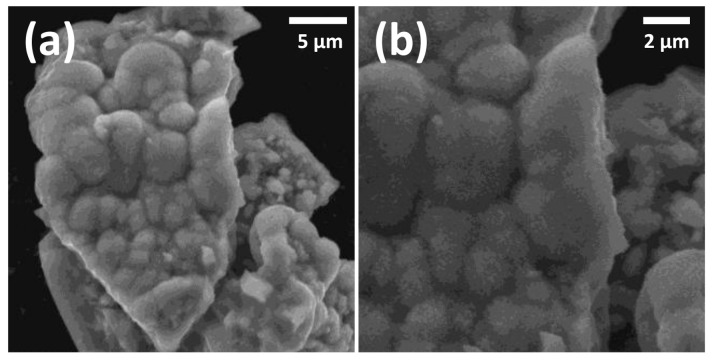
SEM image showing morphology of the nanopowders of LiAl(IO_3_)_4_ at two different magnifications. White bars in the figures indicate the scale, respectively (**a**) 5 µm, and (**b**) 2 μm.

A white precipitate of nanoparticles of pure LiAl(IO_3_)_4_ was collected, after a few hours, from an aqueous solution of 4 mmol LiIO_3_ and 1 mmol of Al(NO_3_)_3_·9H_2_O, dissolved in 3 mL of distilled water. The volume of water was kept to the minimum that allowed for the dissolution of the reagents, i.e., the solution was almost saturated. The reaction mixture was subsequently dried in an oven at 70 °C, until the water was completely evaporated. After drying, no residual reagents were found, implying a complete reaction. This was confirmed by EDX analysis (Figure 1), demonstrating the correct stoichiometry and the absence of residual reagents. Within the experimental error, EDX analyses of Al and I content by both weight percent and atomic percent agreed with the expected molar ratio of 1:4. Numerically, the measured Al and I contents were 19.97 at% and 80.03 at%, respectively. As no impurities were detected, the elementary analysis shown in Figure 1 confirmed the composition of the as-prepared LiAl(IO_3_)_4_ samples. The morphology of the synthetized material is shown in the SEM image of Figure 2, exhibiting nano-powder agglomerates with cotton flower shapes.

**Figure 3:**
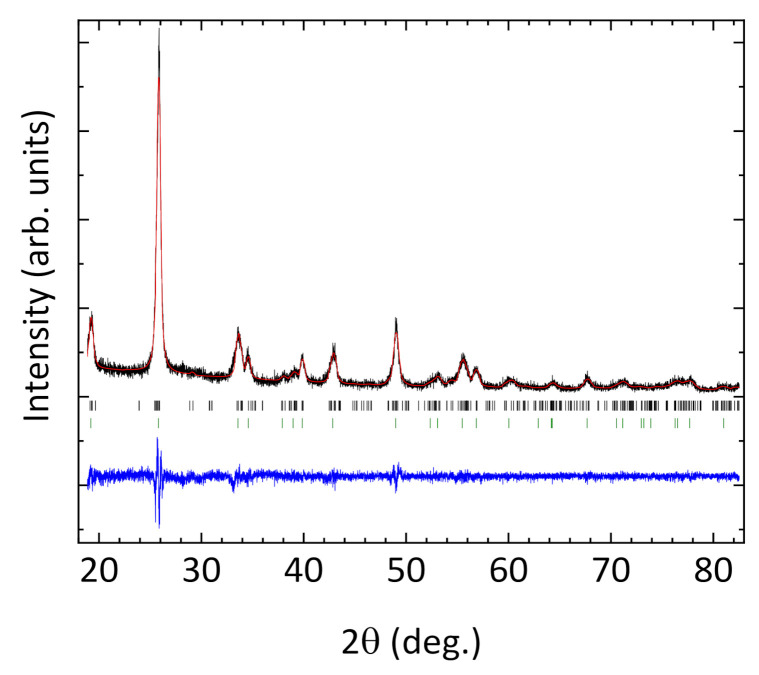
XRD pattern and Rietveld refinement of LiAl(IO_3_)_4_ nanopowders. Black line: experimental data; red line: Rietveld refinement; blue line: residuals; black and green ticks: Bragg reflections calculated using the monoclinic *P*2_1_ and the hexagonal *P*6_3_ space group, respectively. R-factors of the Rietveld refinement: *R*_p_ = 10.41%, *R*_wp_ = 14.28%, $\chi^{2}$ = 1.29.

Figure 3 shows the X-ray diffraction patterns of LiAl(IO_3_)_4_ nanoparticles. To the best of our knowledge, the only mention of LiAl(IO_3_)_4_ in the literature is in Ref. [42]. A hexagonal structure (space group *P*6_3_) was proposed, with lattice constants a=5.585 Å and c=4.945 Å. This structure was derived from the structure of LiIO_3_, assuming that Li and Al atoms in LiAl(IO_3_)_4_ randomly occupy the atomic positions of Li in LiIO_3_ with a 0.5 occupancy factor. However, this structure does not satisfy the stoichiometry of the compound and does not result in the density measured previously [42] and in this work. In addition, we found that a structure based on the *P*6_3_ space group cannot properly index all the experimental XRD peaks, as demonstrated by the green ticks in Figure 3 for a hexagonal indexation. In fact, an indexation with the hexagonal structure led to a=10.649 Å and c=5.182 Å for the unit-cell parameter, with *a* being almost twice the value previously reported [42]. This suggests that the structure previously reported is not correct and that a lower symmetry structure is needed to account for all experimental diffraction peaks. We found that a monoclinic structure isomorphic to Co(IO_3_)_2_ (space group *P*2_1_) can satisfactorily explain the XRD pattern measured for LiAl(IO_3_)_4_ [43]. This can be seen in Figure 3, where we show the result of a Rietveld refinement and the calculated positions of the different (hkl) reflections. Because of the small nanoparticle size and the significant FWHM of the XRD peaks, a full structural refinement is not yet allowed. Instead, we built a crystal structure based on Co(IO_3_)_2_, assuming two independent positions for Co atoms: one occupied by Li atoms and the other by Al atoms. This structure was subsequently optimized by means of tn Table 1.

**Figure 4:**
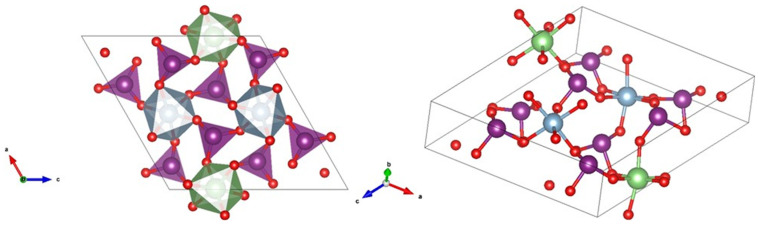
Schematic representation of the crystal structure of LiAl(IO_3_)_4_ from different views. The AlO_6_ and LiO_6_ octahedra are shown (in cyan and green, respectively) as well as the IO_3_ triangular pyramids (in violet).

The atomic positions obtained from DFT were assumed for the Rietveld refinement. They were fixed during the refinement; only the unit-cell parameters, background, and peak-shape parameters were used as fitting parameters. We obtained $a=10.579\left(4\right)$ Å, $b=5.183\left(2\right)$ Å, $c=10.701\left(4\right)$ Å, and $\beta =119.88\left(5\right){}^{\circ}.$ The goodness of the fit refinement parameters were $R_{p}=10.41\%$, $R_{\mathrm{wp}}=14.28\%,$ and $\chi^{2}=1.29$. As illustrated in Figure 4, the Li^1+^ and Al^3+^ cations are coordinated to six O atoms and form slightly distorted octahedral units, which are connected by IO_3_ triangular pyramids. This coordination for I^5+^ ions is typical of metal iodates, being related to the existence of a lone pair of electrons of the I atom in the IO_3_ molecule. In the proposed crystal structure, each octahedral unit is surrounded by six IO_3_ groups. Regarding the nanoparticle size leading to the XRD peak-broadening, the Le Bail global fitting procedure was implemented by assuming a spherical morphology and the absence of any strain, and by considering the Lorentzian and Gaussian parts of the peak shape. The nanocrystal size thus derived at 22.7 nm is in good agreement with the value at ≈22.9 nm obtained by the Williamson-Hall method [44,45,46].

### 3.2. FTIR, UV-Vis and SHG Measurements

**Figure 5:**
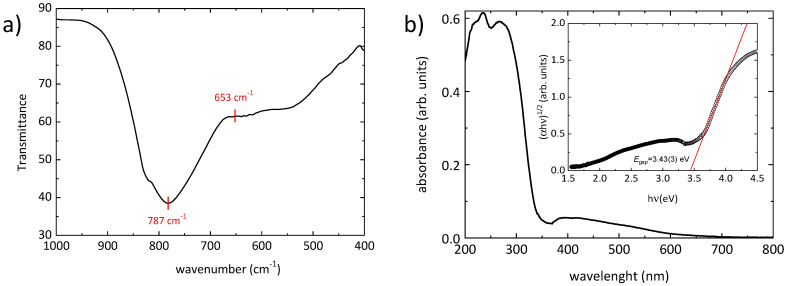
(**a**) FTIR transmission spectrum of nanocrystalline LiAl(IO_3_)_4_ in the 1000–400 cm^−1^ range; (**b**) UV-Vis absorption spectrum. The inset in (**b**) shows the determination of the band gap *E*_gap_ by means of a Tauc plot of the absorbance of LiAl(IO_3_)_4_ (indirect band gap).

The results of Fourier-transform infrared spectroscopy (FTIR) measurements are discussed in the following. Figure 5a shows the IR transmission spectrum in the 1000–400 cm^−1^ range. By analogy with other iodates, the two vibrational frequencies of LiAl(IO_3_)_4_ observed at around 660 cm^−1^ and 786 cm^−1^ can be tentatively assigned as the symmetric ($\nu_{1}$ at 780–630 cm^−1^) and anti-symmetric ($\nu_{3}$ at 730–820 cm^−1^) stretching internal modes of the pyramidal IO_3_^−^ ion, respectively. These values are compatible with the equivalent modes of Al(IO_3_)_3_ [47], La(IO_3_) [22], Zn(IO_3_)_2_ [26], and LiZn(IO_3_)_3_ [37], among other iodates.

As shown in the following discussion, according to our DFT calculations, the band gap of LiAl(IO_3_)_4_ is indirect. Thus, the value $E_{\mathrm{gap}}=3.43\left(3\right)$ eV of the band gap wass estimated by extrapolating the linear part of the (*αhν*)^1/2^ curve as a function of the incident phonon energy *hν* (α being the absorbance) in a Tauc plot (Figure 5b) [48].

The optical properties of the particles were explored using UV-Vis absorption spectroscopy. The absorption spectrum of LiAl(IO_3_)_4_ nanoparticles, as shown in Figure 5b, exhibits strong absorbance below 250 nm. An excitonic absorption peak is found at about 275 nm (eV), with an exciton binding energy $E_{B}=1.08$ eV.

**Figure 6:**
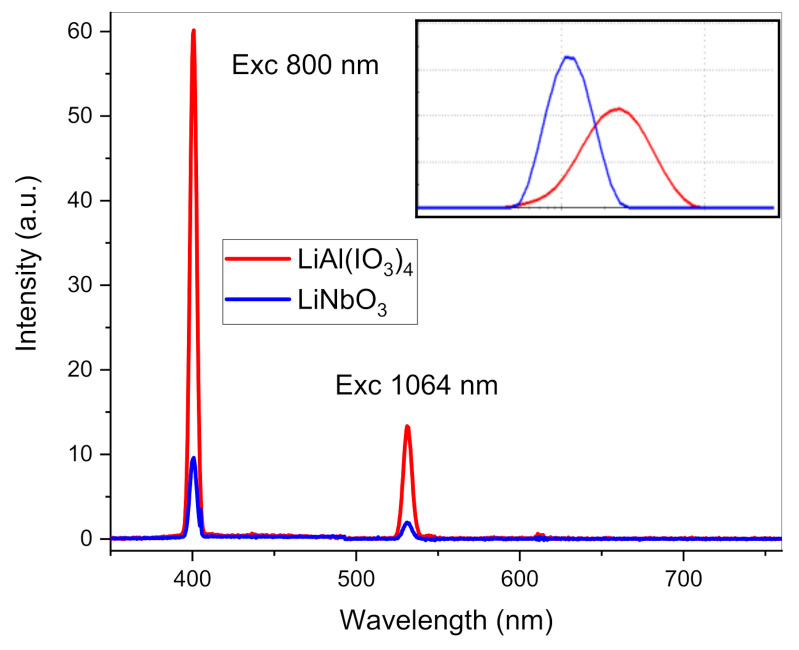
SH scattered intensities collected from a LiAl(IO_3_)_4_ (red curve) and a LiNbO_3_ (blue curve) nanocrystal suspension with similar XRD crystallite sizes and mass concentrations for two excitation wavelengths fixed at 800 nm and 1064 nm. In the inset, the corresponding DLS size distributions in intensity are plotted, showing a higher aggregation state within the supernatant of the LiAl(IO_3_)_4_ suspension.

Finally, because of the non-centrosymmetric space group *P*2_1_ derived above, SHG properties were expected for LiAl(IO_3_)_4_. To get a rapid insight of its SHG efficiency, a suspension was initially prepared at 1 g/L, after sonication of the raw powder in ethanol. After sedimentation for 10 h, drying and weighing of the larger aggregates that had settled down allowed an estimation of the final mass concentration at 0.7 g/L within the supernatant. The corresponding DLS size distribution in intensity is indicated in the inset of Figure 6. The mean size centered at ~255 nm, together with a relatively high PDI value at 0.22, indicated the presence of remaining aggregates whose hydrodynamic diameters were significantly larger than the 23 nm XRD crystallite size derived above. For comparison, a colloidal suspension of LiNbO_3_ nanocrystals, of mean DLS at 120 nm and small PDI value at 0.09, was also prepared at 1 g/L in ethanol. The corresponding mean XRD size at ~30 nm and the synthesis method are described elsewhere [49]. Both suspensions containing LiNbO_3_ and LiAl(IO_3_)_4_ nanocrystals of similar XRD mean size and mass concentration were prepared with the aim of obtaining a rapid comparison of their SHG conversion efficiencies from Second Harmonic (SH) spectroscopy, with the setup fully described in Ref. [50]. Here, for two excitation wavelengths arbitrarily fixed at 800 nm and 1064 nm, the scattered SH intensities are plotted in Figure 6 for the same experimental parameters in the excitation and detection arms. Interestingly, the SHG response of the LiAl(IO_3_)_4_ nanocrystal suspension was found to be above that of the reference LiNbO_3_ material. At this stage, because of the partial aggregation state within the LiAl(IO_3_)_4_ nanocrystal suspension and the eventual presence of a broad size dispersion that would require further TEM imaging, a quantitative assessment of the orientation-averaged second-order susceptibility was difficult to achieve, although promising NLO performances were clearly demonstrated for the iodate modification.

### 3.3. Ab-Initio Calculations

Ab initio simulations were used to calculate the crystal structure of LiAl(IO_3_)_4_. The structure was modeled starting from the isostructural Co(IO_3_)_2_, in which the two independent cobalt atoms were substituted by Li and Al. Subsequently, we performed the optimization of the equilibrium geometry, determining atomic coordinates and lattice parameters. Forces (stresses) on atoms (lattice) were minimized using the Hellmann-Feynman theorem [51,52]. The calculated lattice parameters were $a=10.80\left(5\right)$ Å, $b=5.14\left(4\right)\;$Å, $c=10.89\left(4\right)$ Å, and $\beta =119.00\left(3\right){}^{\circ}$ ($V=528.49\left(9\right)$ Å^3^), which agreed within 1% with those we obtained from the refinements. The calculated atomic positions are summarized in Table 1. The molar mass of LiAl(IO_3_)_4_ was found to be 1467.07 g mol^−1^, and it has two formula units per cell (Z=2), with a density of ρ=4.61 g cm^−3^. From these calculations, we obtained information on the bond distances. The polyhedron corresponding to the iodate groups IO_3_^−^ exhibited the expected trigonal-pyramidal symmetry, with four different iodine atoms. As can be seen in Table 2, each iodine atom is connected to three oxygen atoms by short bonds. 

The LiO_6_ octahedral units are slightly distorted. The Li^+^ cation is connected by three monodentate bonds with an average I–O length of 2.08 Å and with three additional iodate groups with an average I–O length of 2.14 Å (Figure 4). Similarly, AlO_6_ units are also slightly distorted octahedral units connected to IO_3_ pyramids in a similar way to LiO_6_.

**Figure 7:**
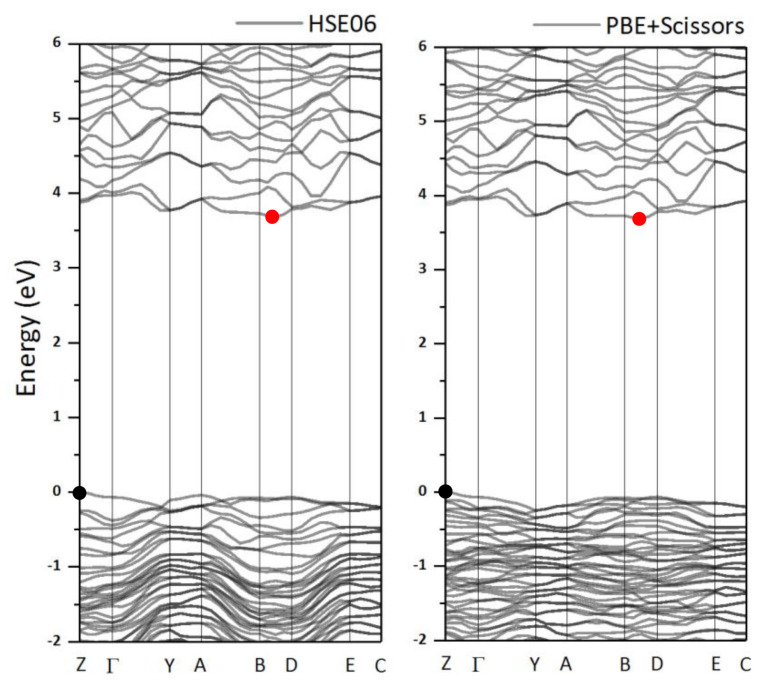
Band structure of LiAl(IO_3_)_4_, as calculated by HDE06 (**left**) and PBE with the scissor parameter (**right**). The red and the black dots represent the top of the valence band and the bottom of the conduction band, respectively.

From DFT calculations, we also determined the electronic band structure of LiAl(IO_3_)_4_, as shown in Figure 7. We found that the material is an indirect semiconductor, with the top of the valence band at the Z point of the Brillouin zone and the bottom of the conduction band between B and D. The calculated band gap was $E_{\mathrm{gap}}=$ 2.43(9) eV or $E_{\mathrm{gap}}=$ 3.68 eV, as calculated by the GGA (PBE) or the HSE06 approach, respectively. The first value underestimated the band gap, a known fact fore PBE functionals, while the hybrid HSE06 functional provided excellent agreement with the calculations. While the band dispersion is well described by PBE, this functional as well as other widely used local functionals, fails in computing the gap value. Therefore, the scissor operator (i.e., the difference between the theoretical value and the most reliable band gap values) was used in this case, which finally gave the same value for the band gap as for HSE06: $E_{\mathrm{gap}}=$ 3.68 eV.

**Figure 8:**
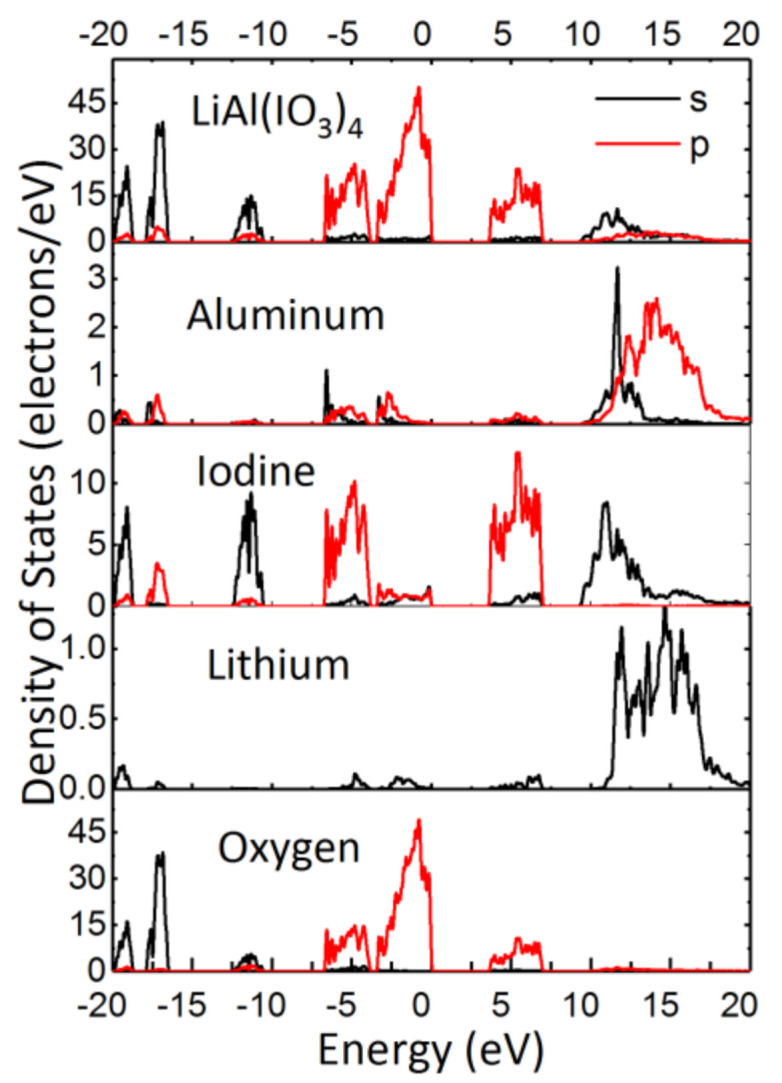
Calculated total distribution of states (DOS) of LiAl(IO_3_)_4_ (top), and partial DOS due to aluminum, iodine, and oxygen atoms in the LiAl(IO_3_)_4_ structure. Black lines: contribution of *s* states; red lines: contribution of *p* states. The calculations were performed using the PBE + scissor method.

The projected density of the states of LiAl(IO_3_)_4_ (PDOS) is shown in Figure 8. The valence band of LiAl(IO_3_)_4_ can be divided into two regions with respect to energy. The first region is in the range of −20 to −10 eV, where the bands are attributed to the I *s* states mixed with some O *s* states, corresponding to a short, strong covalent I–O bond. Contributions of the s and p orbitals of Al are weak in the second energy region, from −7 to 0 eV, since it is dominated instead by the *p* orbitals of I and O, with the latter being the main contribution to the top of the valence band. The bottom of the conduction band, in the range of 0 to 10 eV, is mainly dominated by the *p* orbitals of the iodine atom, with a negligible contribution of the *s* and *p* orbitals of the Al and O *p* states.

The features observed here, from the calculated PDOS of LiAl(IO_3_)_4_, are in a good agreement with those of other non-transition metal iodates widely reported in the literature [23,53,54,55,56,57]. Moreover, as we pointed out in our previous work, if there is no transition metal in the metal iodates, the valence band maxima (VBM) will mainly be dominated by the O 2*p* orbitals, and the conduction band minima (CBM) will mainly be dominated by the I 5*p* orbitals [57]. On the other hand, the partially filled *d* orbitals of transition metal will usually contribute to either the VBM or CBM, thus narrowing the band gap. Based on this fact, the non-transition metal iodates are usually expected to have a relatively large band gap, which can explain the large band gap found here in LiAl(IO_3_)_4_.

**Figure 9:**
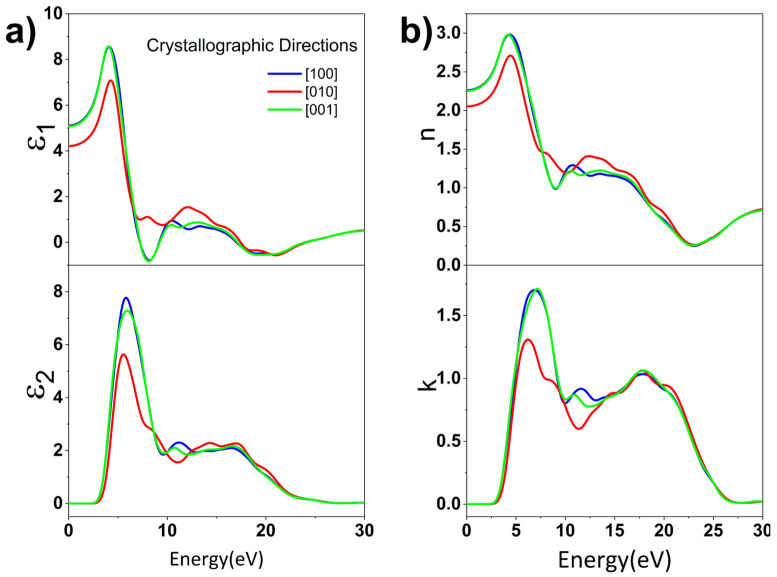
(**a**) Calculated real ($\varepsilon_{1}$) and imaginary ($\varepsilon_{2}$) parts of the dielectric function of LiAl(IO_3_)_4_ as a function of the incident photon energy and (**b**) corresponding real (*n*) and imaginary (*k*) parts of the refractive indexes. The line color is indicated for each crystallographic direction. These calculations have been performed with the PBE + scissor method.

We also studied the linear optical properties of LiAl(IO_3_)_4_, calculating the complex relative permittivity $\varepsilon \left(\omega \right)=\varepsilon_{1}\left(\omega \right)+i\varepsilon_{2}\left(\omega \right)$. The curves of the dielectric function of LiAl(IO_3_)_4_ as a function of the photon energy along the main crystallographic axes, [100], [010], and [001], are shown in Figure 9a. The scissor correction was also added in the computation of optical properties, to avoid underestimation of the band gap due to PBE.

The value of the $\varepsilon_{1}\left(0\right)$ static dielectric constant is about 5, on average. The dispersion curves of the complex refractive index $\underline{n}=n+ik,$ along the main crystallographic directions, are depicted in Figure 9b and show a strong anisotropy between the optical components that are parallel and perpendicular to the [010] direction, in the plane (010) and the direction perpendicular to it [010]. This implies that LiAl(IO_3_)_4_ is a birefringent material, one of the most desirable features for active NLO materials [58,59]. Based on the difference between the refractive indices $n_{100}$ and $n_{010}$ (2.47 (2.26) and 2.22 (2.05) without/with scissor, respectively), the birefringence index of LiAl(IO_3_)_4,_ is $D_{n}=0.21$. Note that zinc iodate (Zn(IO_3_)_2_) and lithium zinc iodate (LiZn(IO_3_)_3_), both crystallizing in the *P*2_1_ space group, have similar birefringence indices at 0.33 and 0.29, respectively [23]. Incidentally, as LiAl(IO_3_)_4_ is colorless, its transparency in the visible domain makes it a potential candidate for quadratic NLO applications and radio communications, in the special case of frequency duplication [60].

Finally, we also computed the components *C*_ij_ of the elastic tensor C of LiAl(IO_3_)_4_, as listed in Table 3. As expected for a monoclinic structure [52], 13 independent values *C*_ij_ exist for LiAl(IO_3_)_4_. In order to be a considered as mechanically stable, a crystal structure at ambient pressure must fulfill the Born criterion, one of whose equivalent formulations is that the eigenvalues of the elastic tensor must all be positive [61]. The eigenvalues of the *C* tensor are shown in Table 4, and they are positive, confirming the stability of the structure.

The computed elastic coefficients are of the same order of magnitude as other NLO materials [62]. Elastic properties such as the bulk (*B*), shear (*G*), and Young’s (*E*) moduli in the Voigt, Reuss, and Hill approximations, and the corresponding Poisson ratio *P*, were calculated and are summarized in Table 5. The calculated bulk modulus *B* of LiAl(IO_3_)_4_ was found to be 28.7–36.1 GPa, indicating that LiAl(IO_3_)_4_ is a fairly compressible material. The obtained bulk modulus is comparable to that of Co(IO_3_)_2_ (*B* = 29.8 GPa) and Zn(IO_3_)_2_ (*B* = 21.6 GPa), which are structurally related to LiAl(IO_3_)_4_ [25,43]. On the other hand, the obtained Poisson ratio is in the lower limits of the typical value for solids [63], and *B/G* is 1.17–126, suggesting that LiAl(IO_3_)_4_ is a brittle material [63].

## 4. Conclusions

We have reported a novel experimental approach for a facile and cost-effective synthesis of likely nontoxic LiAl(IO_3_)_4_ nanocrystals, having potentially high IR NLO performances as exogenous optical biomarkers. The method makes use of a soft-chemistry technique, resulting within a few hours in precipitated nanoparticles from almost saturated solutions of the reagents. No hydrothermal processing is needed. The synthesized samples have been characterized by X-ray diffraction, scanning electron microscopy, EDX, UV-Vis, FTIR, and Second Harmonic spectroscopy. According to XRD results and DFT calculations, the *P*2_1_ monoclinic structure was assigned to LiAl(IO_3_)_4_, instead of to the *P*6_3_ hexagonal space group as previously proposed. Additionally, we also determined an average nanocrystal size at about 23 nm from Le Bail refinements and Williamson-Hall plots. The chemical composition and the expected stoichiometry of the nanocrystals were confirmed by both XRD and EDX. The optical band gap of the nanoparticles was estimated to be 3.43(3) eV, using absorption spectroscopy.

DFT simulations supported our findings about the crystal structure of LiAl(IO_3_)_4._ Theoretical calculations of the band structure showed that LiAl(IO_3_)_4_ is an indirect semiconductor with a calculated band gap *E*_gap_ = 3.68 eV, close to the experimental value. Additionally, the theoretical study of the optical properties suggested that LiAl(IO_3_)_4_ has a birefringence index *D* = 0.21, close to that of the zinc iodate Zn(IO_3_)_2_.

## Figures and Tables

**Table 1:** Atomic positions of LiAl(IO_3_)_4_.

Atom	Wyckoff Position	Atomic Position		
		*x/a*	*y/b*	*z/c*
Al	2a	0.495	0.2942	0.2394
Li	2a	0.0062	0.7938	0.2617
I_1_	2a	0.1832	0.2101	0.5818
I_2_	2a	0.6846	0.2107	0.0857
I_3_	2a	0.154	0.2251	0.087
I_4_	2a	0.6525	0.2241	0.5838
O_1_	2a	0.319	0.023	0.72
O_2_	2a	0.158	0	0.441
O_3_	2a	0.038	0.093	0.5989
O_4_	2a	0.547	0.072	0.115
O_5_	2a	0.833	0.046	0.228
O_6_	2a	0.342	0.528	0.072
O_7_	2a	0.312	0.067	0.185
O_8_	2a	0.109	0.059	0.922
O_9_	2a	0.0333	0.031	0.1193
O_10_	2a	0.452	0.487	0.376
O_11_	2a	0.176	0.562	0.295
O_12_	2a	0.616	0.085	0.4146

**Table 2:** Interatomic bond distances *d* in the coordination polyhedra of LiAl(IO_3_)_4_.

Atom 1	Atom 2	*d* (Å)	Atom 1	Atom 2	*d* (Å)	Atom 1	Atom 2	*d* (Å)
Al	O_10_	2.020	Li	O_11_	2.066	I_1_	O_3_	1.772
	O_12_	2.021		O_3_	2.073		O_2_	1.785
	O_4_	2.047		O_9_	2.101		O_1_	1.789
	O_7_	2.118		O_2_	2.126			
	O_6_	2.139		O_8_	2.143			
	O_1_	2.180		O_5_	2.156			
I_2_	O_5_	1.810	I_3_	O_7_	1.716	I_4_	O_12_	1.832
	O_4_	1.811		O_9_	1.806		O_10_	1.852
	O_6_	1.853		O_8_	1.829		O_11_	1.870

**Table 3:** Elastic tensor components *C*ij for LiAl(IO_3_)_4_, in GPa, as obtained after a full geometry optimization. The errors obtained via DFT are provided in brackets.

C_11_	C_12_	C_13_	C_15_	C_22_	C_23_	C_25_	C_33_	C_35_	C_44_	C_46_	C_55_	C_66_
78(3)	13.4(6)	23.6(7)	−1(1)	35(1)	21(1)	−4(1)	96(3)	1.6(2)	30.8(5)	−2.1(2)	41.3(5)	21(2)

**Table 4:** Eigenvalues of the elasticity matrix C of LiAl(IO_3_)_4_, in GPa.

λ_1_	λ_2_	λ_3_	λ_4_	λ_5_	λ_6_
19.61	26.45	31.19	42.3	61.58	118.96

**Table 5:** Bulk (*B*), shear (*G*) and Young’s (*E*) moduli, and the Poisson ratio for LiAl(IO_3_)_4_.

	Voigt	Reuss	Hill
*B* (GPa)	32.4	36.1	28.7
*G* (GPa)	26.5	28.6	24.4
*E* (GPa)	62.5	67.9	57.0
Poisson ratio	0.18	0.19	0.17

## Data Availability

Data from experiments and calculations are available at request from the authors.
